# Complete Genome Sequence of a Canadian Strain of Raoultella planticola with Metal and Antimicrobial Resistance Genes

**DOI:** 10.1128/MRA.00415-21

**Published:** 2021-06-24

**Authors:** Mingsong Kang, John Chmara, Sohail Naushad, Hongsheng Huang

**Affiliations:** a Canadian Food Inspection Agency, Ottawa Laboratory—Fallowfield, Ottawa, Ontario, Canada; University of Maryland School of Medicine

## Abstract

Raoultella planticola is a Gram-negative opportunistic bacterial pathogen associated with hospital-acquired infections in humans. Here, we report the complete genome sequence of one Raoultella planticola strain isolated from Canadian wastewater treatment facilities containing one chromosome and four plasmids with four antimicrobial resistance (AMR) genes and four metal resistance gene clusters.

## ANNOUNCEMENT

Raoultella planticola is commonly found in environmental habitats (1, 2) and is considered an emerging pathogen due to recent reports of severe infections (3–5). A large number of *R. planticola* isolates with antimicrobial resistance (AMR) have been reported (6, 7), causing concerns regarding antibiotic treatment for *R. planticola* infections. This article reports the complete genome sequence of *R. planticola* strain HH15, containing multiple metal and AMR genes and isolated in 2010 from a pretreated sample in a Canadian wastewater treatment facility. The strain was isolated using a procedure for testing Escherichia coli by preenrichment in lauryl sulfate tryptose broth (35°C, 24 h), selective enrichment in E. coli broth (45°C, 24 h), and isolation using Levine’s eosin methylene blue agar (35°C, 24 h), followed by biochemical identification using an API-20E biochemical test (bioMérieux Canada, Inc.) (8).

Genomic DNA was extracted from an overnight aerobic culture in tryptic soy broth using a NanoBind CBB big DNA kit (Circulomics, USA), followed by treatment with a short-read eliminator XS kit (Circulomics) according to the manufacturer’s instructions. Illumina sequencing was conducted by library preparation using an Illumina DNA prep kit (Illumina, USA), and the library of 300-bp paired-end reads was sequenced on the Illumina MiSeq platform, with a total output of 1,809,114 reads obtained, followed by filtration and trimming with BBTools v38.87 (9). Nanopore sequencing was performed as follows. A MinION library was generated using the 1D native barcoding genomic DNA protocols (EXP-NBD103 and SQK-LSK108; Oxford Nanopore Technologies, UK) without shearing. The library was sequenced using a FLO-MIN106 (R9.4.1) flow cell and a MinION 1B device, yielding a total of 18,183 reads (*N*_50_, 35,937 bp), followed by base calling using Guppy v3.4.5, trimming using Porechop v0.2.3, and filtration using Filtlong v0.2.0 (10). Hybrid assembly of Illumina paired-end and MinION reads was performed using Unicycler v0.4.5 (11). The circularity of the genome was determined using Unicycler v0.4.5, and the genome was rotated using *dnaA* as the starting point. The sequencing coverage depth was determined and assessed using Minimap2 v2.17 (12) and SAMtools v1.10 (13), respectively (9, 10). Gene predictions and annotations were performed using the NCBI Prokaryotic Genome Annotation Pipeline (PGAP) (14). AMR genes were identified using ABRicate v1.0.1 with the NCBI database (accessed 19 April 2020). Potential metal resistance genes were identified by examining the annotations assigned by PGAP. The plasmids were identified by mlplasmids v1.0.0 using Klebsiella pneumoniae as the species model (15), and the prophage sequences were analyzed using PHASTER (16). Default parameters were used for the pipelines except where otherwise noted.

The HH15 genome contains a single chromosome and four plasmids with a total length of 5,938,648 bp, a protein count of 5,421, a GC content of 55.38%, 85 tRNAs, 3 intact prophages, 4 AMR genes (*oqxB9*, *oqxA6*, *bla*_PLA-2a_, and *fosA*), and 4 potential heavy metal resistance gene clusters for mercury, silver, copper, and arsenic. The median total length (5.85103 Mb), the median protein count (5,371), and the median GC content (55.5%) of the Raoultella planticola genome assemblies in GenBank (the data were summarized on 26 February 2021) are similar to those of Raoultella planticola HH15.

### Data availability.

The whole-genome sequence of strain HH15 has been deposited at GenBank under accession number GCA_011290675.2. The version described in this paper is the second version. The MinION and MiSeq base-called fastq files are available in the NCBI Sequence Read Archive under accession numbers SRR12464722 and SRR13628064, respectively.
